# A novel homozygous frame-shift mutation in the *SLC29A3* gene: a new case report and review of literature

**DOI:** 10.1186/s12881-019-0879-7

**Published:** 2019-08-29

**Authors:** Sadaf Noavar, Samira Behroozi, Taraneh Tatarcheh, Farshid Parvini, Majid Foroutan, Hossein Fahimi

**Affiliations:** 1 Pharmaceutical Sciences Research Center, Tehran medical Sciences, Islamic Azad University, Tehran, Iran; 2 Department of Biology, Faculty of Basic Sciences, Semnan University, Semnan, 35131-19111 Iran; 3 Department of Internal Medicine, Semnan University of Medical Sciences, Semnan, Iran; 4 Department of Genetics, Faculty of Advanced Science and Technology, Tehran Medical Sciences, Islamic Azad University, Tehran, 1916893813 Iran

**Keywords:** *SLC29A3*, Frame-shift mutation, SLC29A3-disorder, Iran

## Abstract

**Background:**

The *SLC29A3* gene, encoding a nucleoside transporter protein, is found in intracellular membranes. Based on the literatures, mutations in this gene cause a wide range of clinical manifestations including H syndrome, pigmented hypertrichosis with insulin dependent diabetes, Faisalabad histiocytosis, and dysosteosclerosis. However, all these disorders with their different names and terminologies are actually the same entity termed H syndrome.

**Case presentation:**

We report four *GJB2* and *GJB6* negative deaf patients from two Iranian related families who present the associated symptoms of SLC29A3-disorder. Whole Exome Sequencing (WES) using Next Generation Illumina Sequencing was used to enrich all exons of protein-coding genes as well as some other important genomic regions in one of studied patients. A novel homozygous frame-shift mutation c.307-308delTT (p.Phe103fs) in exon 3 of *SLC29A3* gene was identified in a 35 years old man with profound hearing loss, camptodactyly, rheumatoid arthritis and delayed puberty without any skin changes, short stature and insulin dependent diabetes mellitus. The mutation found was also confirmed by Sanger sequencing in other studied patients and their healthy parents. In compared to proband, however the clinical manifestations of these patients were different, indicating variable expressivity of mutant *SLC29A3* gene as well as possible involvement of other modifier genes.

**Conclusion:**

The present study uncovered a rare novel homozygous frame-shift mutation c.307-308delTT in *SLC29A3* gene of four related patients with various manifestation of SLC29A3-disorder. Such studies can help to conduct genetic counseling and subsequently, prenatal diagnosis more accurately for individuals at the high risk of these types of genetic disorders.

## Background

The *SLC29A3* gene, also called ENT3 (equilibrative nucleoside transporter 3), encodes a nucleoside transporter protein. The ENT3 protein is found in intracellular membranes, especially in lysosomal and mitochondrial membranes [1, 2]. Because of wide spread biochemical roles of nucleoside molecules, any disruption in metabolism and trafficking of nucleosides could result in various abnormal phenotypes.

Based on the literatures, the mutations in the *SLC29A3* gene cause a wide range of different clinical manifestations including H syndrome (defined by cardiac anomalies, camptodactyly, short stature, hypergonadotropic hypogonadism, hepatosplenomegaly, scrotal masses, growth retardation, and sensorineural hearing loss [3–7]), pigmented hypertrichosis with insulin dependent diabetes (PHID) (characterized by hyperpigmented and hypertrichotic skin, hepatosplenomegaly, hypogonadism, diabetes mellitus, comptodactyly, clinodactyly, chronic inflammatory syndrome, and growth retardation [8]), Faisalabad histiocytosis (FHC) (defined by deafness, growth retardation, hypogonadism, camptodactyly, and Rosai Dorfman disease [9, 10]), and dysosteosclerosis (DSS) [8, 11, 12] (characterized by hyperpigmented skin, frontal bossing, mid-face hypoplasia, short stature, sclerotic platyspondyly, otosclerosis, and compression of CNS and cranial nerves [11]). However, it is worth mentioning that all these disorders, with their different names and terminologies, are actually the same entity termed H syndrome. For this reason, their description as different entities is misleading. As outlined above, a number of phenotypes including patches and plaques of hyperpigmented and hypertrichotic skin, short stature, camptodactyly, hearing problems, myelofibrosis, hepatosplenomegaly, pulmonary stenosis, pericarditis, diabetes, and hypogonadism [7, 11, 13] are common among H syndrome affected patients but not all phenotypes are seen in all patients. In this report, we describe a novel homozygous frame-shift mutation in *SLC29A3* gene and its related phenotypes in two Iranian related families.

## Case presentation

**Fig. 1:**
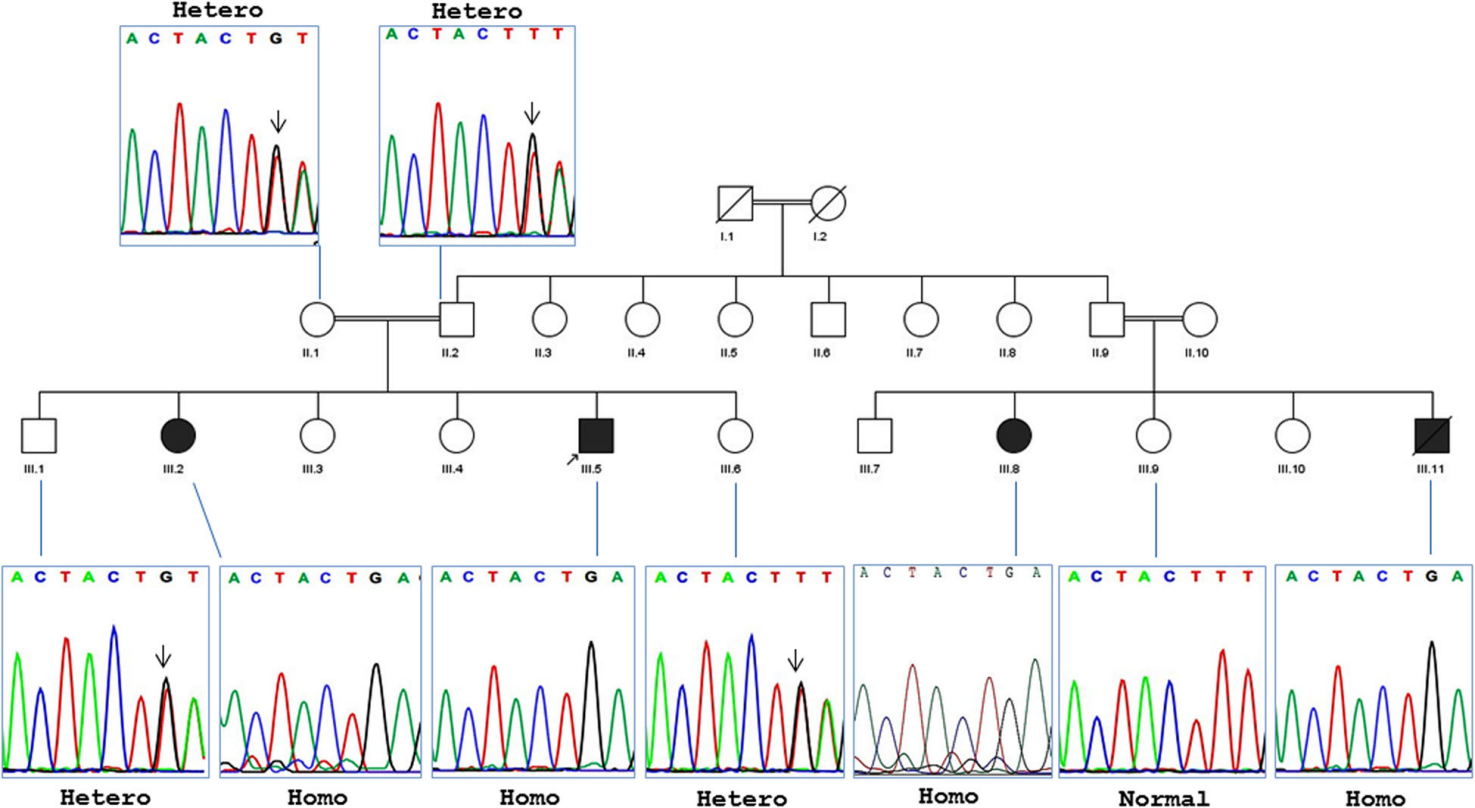
Pedigree and segregation analysis of the SLC29A3 gene mutation (c.307-308delTT). All four patients (III.2, III.5, III.8, and III.11) are homozygote mutants, whereas parents of proband (II.1 and II.2) are heterozygote. Furthermore, sequence analysis of the three normal siblings of the patients in two families revealed that two of them (III.1 and III.6) are heterozygote and one (III-9) is normal

**Fig. 2:**
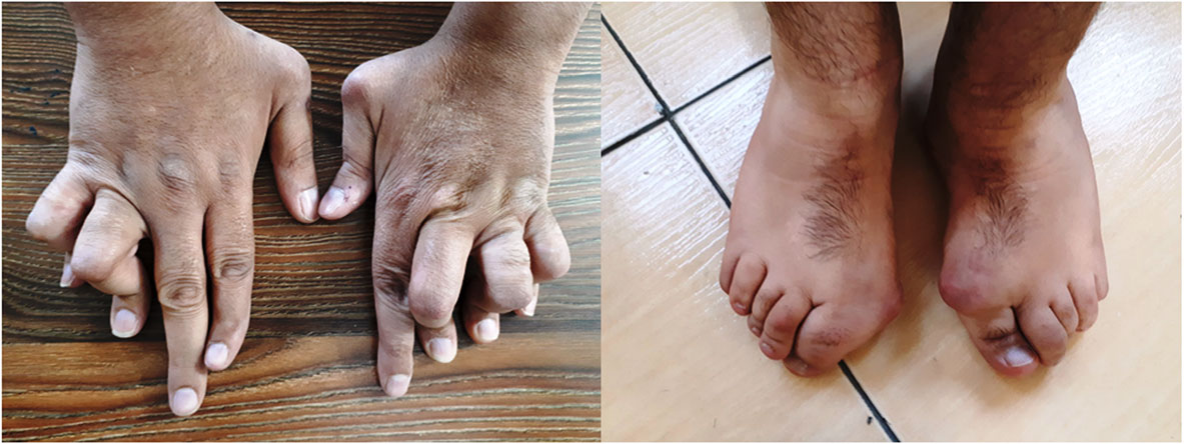
The hands and feet of patient III.5 which show camptodactyly phenotype

The studied families are of Iranian origin located in Semnan province (central Iran). As shown in Fig. 1, four affected individuals are included in two related families (a brother and a sister from each family), with two common ancestors. All four patients were born at term, with normal health indicators and metrics, following an uneventful pregnancy. Their ages ranged between 35 and 46 years. The clinical and demographic data of the patients are summarized in Table 1. In addition, camptodactyly phenotype in patient III-5 is depicted in Fig. 2.

**Table 1:** Summary of clinical and demographic findings

	Family 1	Family 2		
	Patient III-2	Patient III-5	Patient III-8	Patient III-11^a^
Sex	Female	Male	Female	Male
Age (years)	46	35	37	41
Height (cm)	170	178	167	180
Age of onset (years)^b^	15	6	7–8	5
Hearing loss	Mild	Profound	Profound	Profound
Camptodactyly	+	+	+	+
ESR (mm/h)	70–110	45	No data	No data
Polyclonal gammopathy	Severe	Mild	Mild	–
Skin changes	No	No	No	No
IDDM	–	–	+	+
Arthropathy	+	+	+	+
Delayed puberty	Yes	Yes	No	No
CRP mg/dL	30–90	34	No data	No data
Rheumatoid arthritis	+	+	+	+
Lymph nodes	Generalized Lymphoadenopathy	No	No	No

*ESR* erythrocyte sedimentation rate, *IDDM*  Insulin dependent diabetes mellitus, *CRP* C reactive protein

^a^ The patient III-11 died due to the diabetes

^b^ For clinical manifestations

After obtaining informed consent, the peripheral blood samples were collected from patients and their family members. Genomic DNA was extracted from blood samples by QIAamp DNA Blood Mini Kit (Germany) according to the manufacturer’s instructions. At first, the patients were investigated for mutations of *GJB2* gene using ASPCR (Allele Specific PCR) and sequencing followed by screening of two known deletions del(D13S1830) and del(D13S1854) of *GJB6* gene. Since the studied patients were negative for *GJB2* and *GJB6* genes mutations, the patient III-5 was more investigated by Next Generation Sequencing. For this purpose, Whole Exome Sequencing (WES) was used to enrich all exons of protein-coding genes as well as some other important genomic regions. The WES was performed to sequence close to 100 million reads on Illumina HiSeq2000 Sequencer. Generally, the test platform examined > 95% of the targeted regions with sensitivity of above 99%. In this test, point mutations and micro-insertions/deletions as well as duplications (< 20 bp) can be simultaneously detected. Analysis of the sequencing results was performed using BWA aligner [14], annovar [15] and GATK [16] open access software as well as public databases ExAC, gnomAD, Kaviar (~Known VARiants) and GME. Additionally, ACMG guidelines and local population database with more than 1500 unrelated individuals (BayanGene) were used. Standard bioinformatics tools used were as follows: CADD_phred, REVEL, MCAP, SIFT, Polyphen, LRT, MutationTaster, and MutationAssessor. Furthermore, to confirm the novel mutation found in the *SLC29A3* gene, PCR and sequencing were performed for patients and their normal family members using following primers: F-5′ CAGTCCATGGGCAGAAGTGT 3′ and R- 5′ TCGCCTACCTGTTGACAAGC 3′ (PCR product: 401 bp). Finally, the Sanger sequencing data was analyzed by Chromas software.

**Fig. 3:**
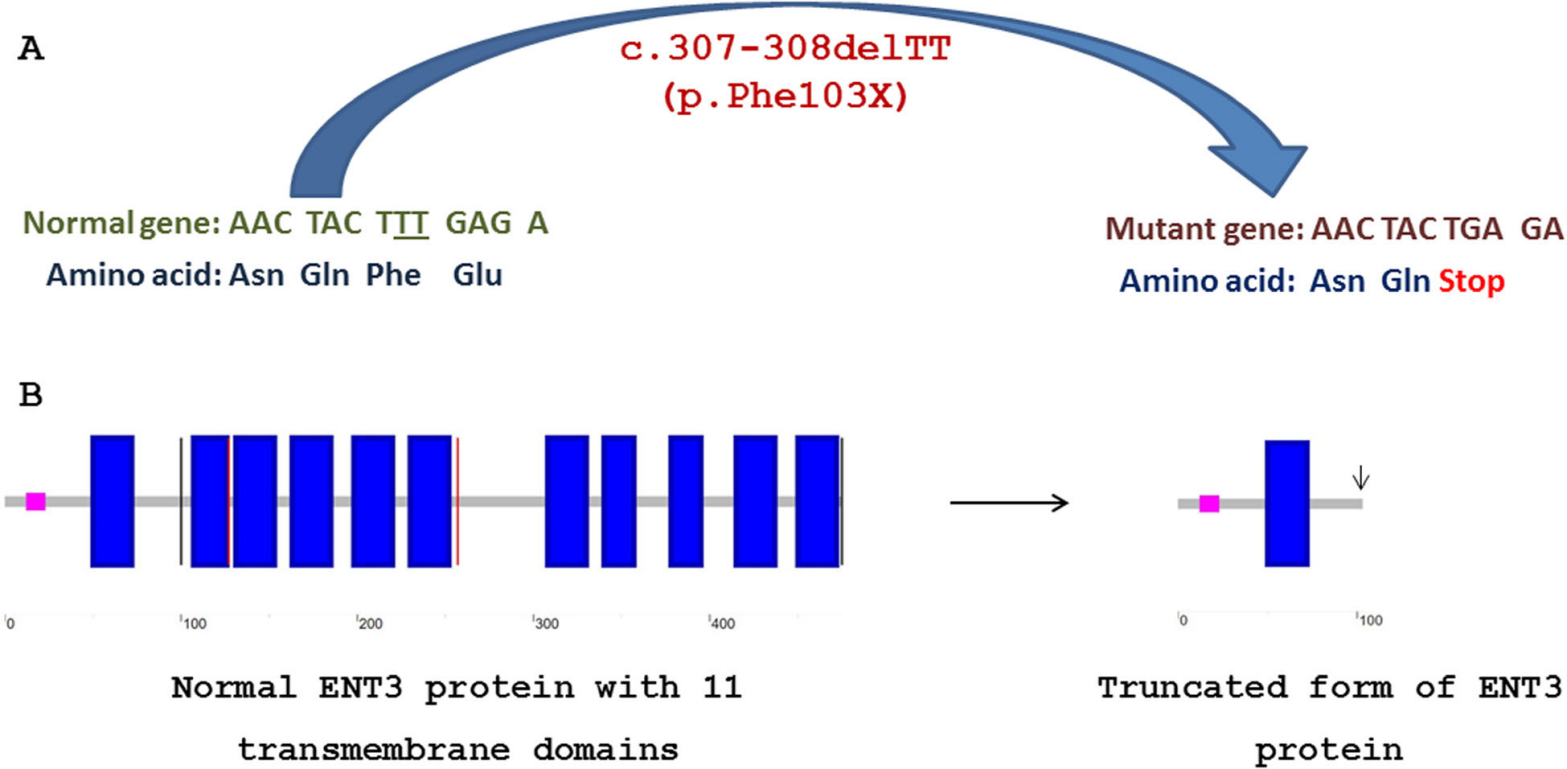
Schematic illustration for loss of 10 out of 11 transmembrane domains of ENT3 protein as a consequence of c.307-308delTT (p.Phen103fs) frame-shift mutation in *SLC29A3* gene. **a** The normal sequence of the gene and the corresponding amino acid sequence have shown on the left. Two deleted thymidines are underlined. The mutated sequence and the corresponding truncated protein are depicted on the right. **b** Ten out of 11 transmembrane domains of ENT3 protein are deleted due to frame-shift mutation c.307-308delTT (p.Phen103fs)

Based on the results, all four analyzed patients were negative for *GJB2* mutations and two common large deletions of *GJB6* gene. Subsequently, the NGS analysis of proband (patient III-5) revealed one deleterious homozygous mutation in *SLC29A3* gene. This homozygous deletion includes two thymidine nucleotides (c.307-308delTT) of exon 3 causing a frame-shift mutation that immediately, makes a stop codon (TGA) instead of Phe103 codon (TTT) (*SLC29A3*:NM_001174098:exon3:c.307-308delTT:p.Phe103fs) (Fig. 3). The mutation found, validated by Sanger sequencing.

At the next step, segregation analysis of novel homozygous mutation found was performed for other family members including another three patients (patients III-2, III-8, and III-11). As expected, parents of proband were heterozygote for reported mutation. A similar result was achieved from the parents of patient III-8 (data not shown). Furthermore, the obtained results showed that patients III-2, III-8, and III-11 have the found deleterious mutation. Furthermore, three normal siblings of the patients were also checked for the deleterious mutation 307-308delTT of *SLC29A3* gene. The results clearly revealed that two of them (III-1 and III-6) are heterozygote and one (III-9) is normal homozygote. The corresponding family pedigree with the results of sequence analyses are illustrated in Fig. 1.

## Discussion and conclusion

As previously mentioned, the mutation in *SLC29A3* gene causes a wide range of mild to severe different medical manifestations which are known as the same entity termed H syndrome. To date, more than 22 pathogenic or likely pathogenic mutations have been reported in the *SLC29A3* gene (Table 2). As shown in Table 2, the preliminary report for 308-309delTT (OMIM: 612373.0009) frame-shift mutation, which is resulted in truncated protein has been published in 2010 [10]. However, they found a heterozygous form of this mutation in combination with heterozygous missense mutation Gly437Arg as compound heterozygote, which is led to H syndrome. Here, we have explained the first report of novel homozygous form of this deleterious mutation from two Iranian related families. All four reported patients were results of consanguineous marriages. As previously pointed out, this frame-shift mutation is resulted in premature protein truncation. The truncated ENT3 protein loses its 10 out of 11 transmembrane domains (Fig. 3). Therefore, it seems that this homozygous mutation, as a loss of function mutation, leads to elimination of ENT3 protein function.

**Table 2:** A summary of the reported pathogenic/likely pathogenic mutations of *SLC29A3* gene

Nucleotide change	Genotype	Consequence	(Clinical significance) Main Phenotypes	Family origin	Reference/ (variation ID in ClinVar)
c.243delA	Homo	p.Lys81Asnfs	Pathogenic Nasal infiltration, Pancreatic exocrine deficiency, Insulin-dependent diabetes, Contractures of the fingers, Contractures of the toes, Retroperitoneal fibrosis	Moroccan	[17]
c.308-309delTT	Hetero	p.Phe103Terfs	(Pathogenic) Histiocytosis, Rosai-Dorfman disease	Turkish and Palestinian	[10]
c.300 + 1G > A (IVS2 + 1G > A)^a^	Homo	Splice site		Pakistani	
c.73C > T	–	p.Arg25Ter	(Likely pathogenic) Histiocytosis-lymphadenopathy plus syndrome	–	(ID: 212200)
c.300 + 1G > C	–	Splice site	(Pathogenic) not provided		(ID: 427021)
c.347 T > G	Homo	p.Met116Arg	(Pathogenic) insulin-dependent diabetes mellitus and pigmented hypertrichotic skin lesions	Australian Lebanese	[8]
c.940delT		p.Tyr314ThrfsTer91		Indian	
c.1309G > A		p.Gly437Arg		Pakistani	
c.1330G > T		p.Glu444Ter		North American Caucasian	
c.1346C > G		p.Thr449Arg		Australian Lebanese	
c.479G > A	–	p.Trp160Ter	(Pathogenic) Histiocytosis-lymphadenopathy plus syndrome	–	(ID: 573984)
c.607 T > C	Hetero	p.Ser203Pro	(Pathogenic) Dysosteosclerosis	–	[11]
c.1157G > A		p.Arg386Gln			
c.1346C > G	Homo	p.Thr449Arg			
c.714_715invTG	–	p.Val239Ile	(Likely pathogenic) Histiocytosis-lymphadenopathy plus syndrome	–	(ID: 300363)
c.1001A > G	–	p.Asn334Ser	(Likely pathogenic) Histiocytosis-lymphadenopathy plus syndrome	–	(ID: 300368)
c.1045delC	Homo	p.Leu349Serfs	(Pathogenic) Hyperpigmentation, Fixed flexion contractures of proximal interphalangeal joints, Hallux valgus, Prominent gynecomastia, histiocytic and dendritic infiltrate	Bulgarian	[18]
c.1087C > T		p.Arg363Trp		Spanish	[4]
c.1088G > A		p.Arg363Gln		Arab	
c.1228C > T	–	p.Gln410Ter	(Pathogenic) Histiocytosis-lymphadenopathy plus syndrome	–	(ID: 130338)
c.1279G > A	Hetero	p.Gly427Ser	(Pathogenic) seronegative polyarthritis, hypogonadotropic hypogonadism, hearing loss, Proptosis, Arthropathy, Camptodactyly, Delayed puberty, Polyclonal gammopathy	Arab	[7]
c.307-308delTT	Homo	p.Phe103Terfs	(Pathogenic) Hearing loss, Camptodactyly, Polyclonal gammopathy, Arthropathy, Delayed puberty, Rheumatoid arthritis	Iranian	This report

^a^*IVS* InterVening Sequence (i.e. an intron)

As shown in Table 1, some of the common manifestations have been seen in our patients. Nonetheless, some of the phenotypes have not seen or were different among patients of two studied families. For example, although the short stature is reported in mostly reports for H syndrome [7, 11, 13], all four patients in this report have normal height (167–180 cm), in accordance with two previously reported cases from Morocco [17]. Similarly, and in accordance with some reports [8, 12], all patients in this study have moderate (patient III-2) to profound deafness (patients III-5, III-8, and III-11). Deafness has also been reported in other previous reports [4, 18], which is in accordance with our observations. Although these reported patients showed other manifestations including, hyperpigmentation, hypertrichosis, fixed flexion contractures of proximal interphalangeal joints, Hallux valgus and fixed flexion contractures of toe joints. However, the hearing loss has not been reported in originally reported PHID (other form of H syndrome) patients [19, 20]. Delayed puberty was observed in patients of family 1 (Patients III-2 and III-5). This observation is in line with those reported by Cliffe et al. (2009) and Hussain et al. (2009) [8, 19].

In accordance with other reports [7, 11], all four studied patients were suffered from camptodactyly. While the normal ESR range is 0–22 mm/h for men and 0–29 mm/h for women, a high ESR was observed in two patients III-2 and III-5. On the other words, it seems that the high levels of CRP in patients III-2 and III-5 could because of rheumatoid arthritis in these patients (normal CRP level is below 3.0 mg/L). Although, there was no data for ESR and CRP levels of patients III-8 and III-11, clinical symptoms of the rheumatoid arthritis were also confirmed by a rheumatologist in these patients. This result is in agreement with clinical symptoms of a previously reported patient [7]. While, the severe polyclonal gammopathy was observed in patient III-2, a mild representation was observed in patients III-5 and III-8 indicating variable expressivity of mutant *SLC29A3* gene. Polyclonal gammopathy could be due to the elevated production of immunoglobulins by plasma cells [21]. Unlike the monoclonal gammopathy, polyclonal gammopathy is associated with a nonmalignant condition. In the present study, patient III-2 has been shown generalized lymphadenopathy. This observation is similar to previously reported lymphadenopathy in mutated *SLC29A3* patients [5, 7, 8, 10, 11, 20]. Corresponding to the previously report from Morocco [17], despite the hyperpigmented/hypertrichotic skin is the common feature in *SLC29A3* mutants [11], none of the patients reported in this paper have skin changes. Similarly, although mutation in *SLC29A3* gene mostly leads to diabetes [7, 11, 22], we observed this phenotype only in the patients of the family 2 (patients III-8 and III-11) showing the variable expressivity and eventually involvement of other genes. Similar observation has been conducted by Spiegel et al. (2010) [7]. All four patients were suffered from arthropathy and rheumatoid arthritis. This is in accordance with several previously reported cases [7, 23].

In conclusion, this report shows that a homozygous frame-shift mutation c.307-308delTT results in a complex of clinical manifestations of H syndrome. In addition to allelic heterogeneity, it seems that these various clinical symptoms clearly confirm the pleiotropic effects and variable expressivity of *SLC29A3* gene as well as possible involvement of other genes. On the other words, the obtained results showed that whole-exome sequencing is a useful method discovering genes involved in human genetic diseases.

## Data Availability

The datasets generated during and/or analyzed during the current study are available from the corresponding authors on reasonable request.

## Abbreviations

ASPCR: Allele Specific PCR
CRP: C reactive protein
DSS: Dysosteosclerosis
ENT3: Equilibrative nucleoside transporter 3
ESR: Erythrocyte sedimentation rate
FHC: Faisalabad histiocytosis
GJB2: Gap junction beta-2 protein
GJB6: Gap Junction Beta 6 protein
IDDM: Insulin dependent diabetes mellitus
NGS: Next generation sequencing
PHID: Pigmented hypertrichosis with insulin dependent diabetes
SHML: Histiocytosis with massive lymphadenopathy
SLC29A3: Solute Carrier Family 29 Member 3
WES: Whole Exome Sequencing
